# A Scoping Review of Geriatric Release and the Later Life Impacts for Incarcerated Older Adults

**DOI:** 10.1093/geroni/igaf122.199

**Published:** 2025-12-31

**Authors:** Samuel Van Vleet, Ryan Steel

**Affiliations:** Miami University, Oxford, Ohio, United States; Miami University, Oxford, Ohio, United States

## Abstract

As the U.S. carceral system grapples with an aging prison population, policies such as compassionate release and geriatric parole are intended to provide pathways for older adults to transition out of incarceration. However, these mechanisms remain underutilized, restrictive, and fraught with systemic barriers. While these programs are designed to provide relief, little is known about the post-incarcerated impacts of reentry for the older adults that are granted this release. This scoping review seeks to address gaps in our understanding of geriatric release programs within the United States by examining both the broader challenges of reentry and the unique barriers that older adults face. Older adults navigating reentry face barriers common to the broader formerly incarcerated population, such as parole compliance, recidivism risk, transportation, and healthcare access. These challenges are heightened by aging-related issues like chronic health conditions, limited employment opportunities, and social isolation. Beyond the individual, these difficulties place additional strain on families and communities that are essential to reintegration. Findings highlight a critical gap in research on post-release outcomes for older adults and a need for stronger evaluation of geriatric release policies. As mass incarceration’s long-term consequences unfold, this paper calls for urgent policy reform, targeted reintegration support, and stronger collaborations between gerontology and criminal justice advocacy. Without meaningful intervention, the aging prison population will face worsening health and social disparities, exacerbating existing inequities and straining already limited resources.

